# Stellate Cells Aid Growth-Permissive Metabolic Reprogramming and Promote Gemcitabine Chemoresistance in Pancreatic Cancer

**DOI:** 10.3390/cancers13040601

**Published:** 2021-02-03

**Authors:** Manoj Amrutkar, Ivar P. Gladhaug

**Affiliations:** 1 Department of Pharmacology, Institute of Clinical Medicine, University of Oslo, 0316 Oslo, Norway; 2 Department of Hepato-Pancreato-Biliary Surgery, Institute of Clinical Medicine, University of Oslo, 0318 Oslo, Norway; i.p.gladhaug@medisin.uio.no; 3 Department of Hepato-Pancreato-Biliary Surgery, Oslo University Hospital Rikshospitalet, 0424 Oslo, Norway

**Keywords:** pancreatic cancer, pancreatic stellate cell, metabolic reprogramming, gemcitabine chemoresistance

## Abstract

**Simple Summary:**

The great majority, more than 90%, of patients with pancreatic ductal adenocarcinoma (PDAC) die within less than five years after detection of the disease, despite recent treatment advances. The poor prognosis is related to late diagnosis, aggressive disease progression, and tumor resistance to conventional chemotherapy. PDAC tumor tissue is characterized by dense fibrosis and poor nutrient availability. A large portion of the tumor is made up of stromal fibroblasts, the pancreatic stellate cells (PSCs), which are known to contribute to tumor progression in several ways. PSCs have been shown to act as an alternate energy source, induce drug resistance, and inhibit drug availability in tumor cells, however, the underlying exact molecular mechanisms remain unknown. In this literature review, we discuss recent available knowledge about the contributions of PSCs to the overall progression of PDAC via changes in tumor metabolism and how this is linked to therapy resistance.

**Abstract:**

Pancreatic ductal adenocarcinoma (PDAC), also known as pancreatic cancer (PC), is characterized by an overall poor prognosis and a five-year survival that is less than 10%. Characteristic features of the tumor are the presence of a prominent desmoplastic stromal response, an altered metabolism, and profound resistance to cancer drugs including gemcitabine, the backbone of PDAC chemotherapy. The pancreatic stellate cells (PSCs) constitute the major cellular component of PDAC stroma. PSCs are essential for extracellular matrix assembly and form a supportive niche for tumor growth. Various cytokines and growth factors induce activation of PSCs through autocrine and paracrine mechanisms, which in turn promote overall tumor growth and metastasis and induce chemoresistance. To maintain growth and survival in the nutrient-poor, hypoxic environment of PDAC, tumor cells fulfill their high energy demands via several unconventional ways, a process generally referred to as metabolic reprogramming. Accumulating evidence indicates that activated PSCs not only contribute to the therapy-resistant phenotype of PDAC but also act as a nutrient supplier for the tumor cells. However, the precise molecular links between metabolic reprogramming and an acquired therapy resistance in PDAC remain elusive. This review highlights recent findings indicating the importance of PSCs in aiding growth-permissive metabolic reprogramming and gemcitabine chemoresistance in PDAC.

## 1. Introduction

Pancreatic ductal adenocarcinoma (PDAC), commonly referred to as pancreatic cancer (PC), comprises more than 85% of all pancreatic tumors. PDAC is a highly malignant tumor with a notoriously dismal prognosis [1] and ranks among the leading causes of cancer-related deaths in the Western world [2]. The overall five-year survival rate of merely 7% for all stages of the disease is among the lowest of all solid tumor types [3]. Despite certain recent treatment advances such as neoadjuvant treatment followed by surgery and combination chemotherapies using FOLinic acid, 5-Fluorouracil, IRINotecan, and Oxaliplatin (FOLFIRINOX) and/or gemcitabine, the overall prognosis of PDAC patients remains poor [4,5,6].

The dismal clinical outcomes have been linked to the presence of both intrinsic (de novo) and acquired resistance to the existing therapeutics, especially against gemcitabine. Since 1997, gemcitabine has been the gold standard of care for all stages of PDAC [7,8]. One of the main challenges of PDAC treatment lies in the fact that cytotoxic drugs may achieve good results in preclinical test models, however, they generally fail to do so when tested clinically. The main culprit is the presence of an exceedingly prominent stroma in PDAC, which acts as a mechanical barrier to drug delivery, and imparts drug resistance through various mechanisms that are still only partially characterized and understood [9,10]. In addition, the presence of high inter- and intra-tumor heterogeneity is another hallmark of PDAC [11]. Accumulating evidence indicates that morphological heterogeneity in PDAC is accompanied by a marked genetic heterogeneity [12,13]. Tumor heterogeneity results in significant variability in treatment outcomes for existing therapeutics and makes the development of new therapeutics challenging.

Because the existing treatment modalities beyond surgical resection seem to be largely ineffective in PDAC, several different aspects of pancreatic tumor biology are being investigated to identify new therapeutic strategies and targets. One of these aspects is the altered metabolism that prevails in cancers. Metabolic alterations within a tumor have long been recognized as a hallmark of cancer in general, however, the exact underlying mechanisms are only recently beginning to be uncovered [14,15,16,17]. Growing evidence indicates that metabolism is extensively reprogrammed in PDAC, both at local and systemic levels, and stroma plays a key role in this process [18,19,20]. To date, the nature of the altered metabolism in PDAC, its key drivers, and the overall impact on chemosensitivity remains unclear.

During the last two decades, pancreatic stellate cells (PSCs), also referred to as cancer-associated fibroblasts (CAFs)—the largest cellular component of PDAC tissue—have been well characterized, although important roles are still incompletely understood. PSCs interact closely with the proper malignant epithelial pancreatic cancer cells (PC cells) and with other cell types of the stroma, resulting in a growth permissive environment for pancreatic tumors [21]. Recently, PSCs have also been shown to act as a nutrient supplier to tumor cells and help them survive and proliferate in a challenging PDAC environment [14,22]. Moreover, PSCs are also implicated in the development of therapeutic resistance of PDAC [23,24]. The present review focuses on recent findings that describe the role of PSCs in the metabolic regulation and chemoresistance in PDAC and the subsequent contributions to overall tumor progression.

## 2. Pancreatic Tumor Metabolism

Cancer metabolism, the oldest area of research in cancer biology, is based on the principle that metabolic activities are significantly altered in tumor cells compared to normal cells [25,26]. According to DeBerardinis et al., the most commonly used terms in cancer metabolism are “metabolic reprogramming” and “oncometabolite” [25]. Metabolic reprogramming is defined as alterations of conventional metabolic pathways such that activities are enhanced or suppressed in tumor cells relative to benign cells or tissues as a consequence of tumorigenic mutations and/or other factors, whereas oncometabolite refers to a particular metabolite whose abundance is markedly higher in tumors compared to normal tissue [25]. A classic example of metabolic reprogramming is the “Warburg effect” or aerobic glycolysis [27,28]. In the 1920s, Otto Warburg first observed abnormal energy utilization in cancer cells and noted that these cells constitutively take up glucose and produce lactate regardless of oxygen availability. A similar mechanism was subsequently observed as a general feature of cancer cells and tumors [29]. The process of aerobic glycolysis suggests that even in the presence of sufficient oxygen, the malignant cells prefer to produce adenosine triphosphate (ATP) by glycolysis instead of by oxidative phosphorylation (OXPHOS), which is a less efficient pathway for energy production. According to the Warburg effect, the increased glycolytic phenotype and partial suppression of oxidative metabolism in tumor cells mainly result from mitochondrial dysfunction, i.e., defective mitochondrial OXPHOS [28].

Despite several limitations, the Warburg effect has long been considered a basic principle of energy metabolism in cancer cells. However, several recent studies have revealed inherent contradictions in this principle. For example, unlike the Warburg effect, some tumor cells display high rates of OXPHOS and low glycolysis. Moreover, the two major pathways of energy production in cancer cells, aerobic glycolysis, and OXPHOS, are not mutually exclusive, because each may contribute differentially to ATP production depending upon the tumor microenvironment (TME), normoxia, or hypoxia [30]. In addition, the most intriguing metabolic paradox of how and why tumor cells prefer the production of ATP via the less efficient pathway despite high energy demands for tumor progression remains unresolved. The Warburg effect only partially explains tumor metabolism as it merely focuses on metabolism within cancer cells while neglecting the metabolic interactions between the cancer cells and other components in the microenvironment. The different cellular components in TME produce a metabolic heterogeneity within tumors, with some cells maintaining a glycolytic phenotype while others predominantly utilize OXPHOS [31,32,33]. Interactions between cancer cells and the TME are conducive to the transfer of metabolites from stromal cells to meet the metabolic demands of cancer cells and maintain ATP production in cancer cells [31]. Based on this renewed interest in CAF-cancer cell interactions, a two-compartment model reconsidering tumor metabolism was proposed in 2009, known as the “reverse Warburg effect” [34]. According to the reverse Warburg effect, cancer cells secrete reactive oxygen species (ROS) in TME, which induce oxidative stress in neighboring CAFs. Consequently, the CAFs undergo aerobic glycolysis to produce high energy-fuels such as pyruvate, ketone bodies, fatty acids, and lactate. In turn, these fuels feed OXPHOS in cancer cells, thereby contributing to efficient energy production. In TME, the two distinct metabolic phenotypes glycolysis (the Warburg effect) and mitochondrial OXPHOS (the reverse Warburg effect) co-exist in a metabolic symbiosis and their competition is primarily affected by growth requirements [30,35,36,37].

Following Warburg’s original work, there was little emphasis on cancer metabolism despite the fact that essential hallmarks of cancer are intertwined with an altered cancer cell-intrinsic metabolism, either as a consequence or as a cause. However, during the last decade, there has been a renewed interest in this field, and as such, reprogrammed metabolism is now recognized as a hallmark of cancer [38,39]. There is consensus that pathways for nutrient acquisition and metabolism are reprogrammed in various tumors to meet the energy demands of malignant cells for their survival and proliferation [25,26]. PDAC is a classic example of a tumor with reprogrammed metabolism, in which the pancreatic TME is considered to form a supportive niche by reorganizing various metabolic processes, denoted “metabolic rewiring”. Metabolic rewiring is enhanced in PDAC in order to provide adequate energy and nutrients required for the tumors to grow and pursue their aggressive behavior [14,40].

Frequently mutated oncogenes and tumor suppressors such as KRAS, SMAD4, Myc, and p53 regulate the cellular metabolic state, and KRAS is the main driver of metabolic adaptations within PDAC [14,41]. Acquisition of activating mutations in KRAS is an early event during malignant transformation of nearly all PDACs that influence tumor initiation, progression, and maintenance [42,43,44]. Although KRAS is the critical driver of tumorigenesis, clinically effective pharmacologic KRAS inhibitors have remained unattainable, hence the present standard of care for PDAC still focuses on the use of conventional cytotoxic agents [45]. This highlights the importance of unraveling vulnerabilities of PC cells that can be therapeutically targeted. Indeed, recent studies have revealed a profound activation of metabolic pathways that are downstream of oncogenic KRAS, which holds a promise as a source of targets for new therapeutic strategies [13,46,47]. Activation of these pathways may also be linked to the unique hypovascular, fibrotic microenvironment of PDAC [9]. As a consequence, tumor cells are confronted with hypoxia and limited nutrient availability, both of which are the hallmarks of PDAC. In such a scenario, various cell populations within PDAC adapt early in the tumor development to support a high rate of proliferation and the synthesis of substrates necessary for tumor growth. These changes are broadly categorized as sensing, acquisition, and utilization of the nutrients, and the elimination of toxic by-products [48,49,50].

In response to oxygen deprivation, several metabolic changes occur within PDAC, the process of which is referred to as the “metabolic switch”. It largely involves increased glycolysis, increased amino acid products derived from protein degradation, glycosylation, and fatty acid synthesis, and the recycling and scavenging of the cellular components and metabolites [40,51]. Highly proliferative malignant cells rely heavily on glycolysis, and the use of glucose and glutamine to fulfill their increased energy demands [15]. Similar to several other tumors, PC cells display an increased rate of glycolysis even in the presence of oxygen (the Warburg effect), increased lactate secretion, and thereby promote anabolic glucose metabolism [15,52]. Glutamine is considered an essential amino acid for highly proliferating cells since it supports the synthesis of large amounts of metabolites, and PDAC metabolism is recently shown to be largely glutamine-dependent [15,52,53,54]. Moreover, glutamine supports pancreatic tumor growth through a KRAS-regulated metabolic pathway [55]. Very recently Banh et al. [56] demonstrated axonal–cancer metabolic crosstalk as a critical adaptation in PDAC growth, in which serine released from neurons supports the growth of serine-dependent PDAC cells. Deletion of a cysteine/glutamate transporter Slc7a11 was also recently reported to induce tumor-selective ferroptosis—a form of cell death resulting from the catastrophic accumulation of lipid ROS [57].

Pancreatic tumors also use an intriguing set of scavenging mechanisms for nutrient acquisition, which includes autophagy or “self-cannibalism” and micropinocytosis [15,58,59]. “Autophagy” is a highly conserved cellular catabolic process that mediates the degradation of macromolecules and whole organelles. During episodes of nutrient starvation, the breakdown products generated during autophagy help sustain energy production and the synthesis of cellular building blocks. Hence autophagy is of particular importance for tumor cells to maintain survival in the nutrient-poor microenvironment [60,61]. Furthermore, tumor cells can utilize an additional scavenging pathway, known as “macropinocytosis”. This process, which is upregulated in KRAS-mutant cells including PC cells, involves endocytosis-mediated uptake of extracellular material [59,62]. Both autophagy and macropinocytosis are processes that converge at the lysosome, where cargo is digested by several lysosomal enzymes. Changes in lysosome composition and function have been observed in cancer cells [63]. Moreover, recent findings indicate that increased lysosome biogenesis and function could be integral to the nutrient-scavenging program in PDAC [64,65].

## 3. Pancreatic Stellate Cells—A Key Stromal Component with Several Unknowns

In contrast to early PDAC research typically concentrated on the proper malignant epithelial cancer cells, another major stakeholder, i.e., the PSCs and CAFs, only gained significant attention in recent years [66]. PSCs, the key pro-fibrogenic cells of the pancreas, are considered to play a crucial role in the pathobiology of various pancreatic disorders including pancreatitis and PDAC. The precise origin of PSCs remains unclear; however, they are suggested to be derived from endodermal, mesenchymal, and neuroectodermal origins [21,67]. PSCs can be identified according to their expression of desmin, glial fibrillary acidic protein, vimentin, nestin, and neuroectodermal markers such as nerve growth factor [67].

In the healthy pancreas, resident fibroblasts and quiescent PSCs co-exist to maintain normal gland connective tissue architecture. Quiescent PSCs transform into the activated form in response to injury or tissue damage such as chronic pancreatitis and carcinogenesis [68]. Activated PSCs appear myofibroblast-like, express a high level of α-smooth muscle actin (α-SMA) and lose cytoplasmic lipid droplets containing anti-fibrogenic vitamin A [69,70,71]. Activated PSCs are the primary cellular source of CAFs, and are considered to arise from quiescent PSCs and fibroblasts, through epithelial–mesenchymal transition (EMT), or from bone marrow-derived cells [72]. Interestingly, with repeated or sustained injury, PSCs attain a permanently activated state that is maintained even upon the removal of the primary trigger. Activation of PSCs can occur by both autocrine and paracrine mechanisms. The sustained activation of PSCs is eventually responsible for the development of pathological fibrosis that is often irreversible [67].

Since its first successful isolation in 1998, the role of PSCs in PDAC progression has been extensively characterized, although important aspects are still incompletely understood. Several studies have highlighted the existence of functional heterogeneity among PSCs, consistent with the notion of different subpopulations that can influence tumor progression individually or synergistically, as reviewed elsewhere [73]. The precise nature and the functional landscape of PSCs are not yet fully known. Some of the unresolved questions regarding the nature of PSCs include their exact origin, the transition from quiescent to activated form, and differences between activated PSCs and CAFs. Interestingly, CAFs demonstrate high transcriptional and functional inter- and intra-tumoral heterogeneity, as distinct subpopulations are reported to produce complex effects on the growth and progression of PDAC and on PDAC therapy responses [74,75,76,77,78,79]. Öhlund et al. [74] demonstrated two spatially and functionally distinct CAF subtypes—myofibroblastic (myCAFs) and inflammatory (iCAFs). The myCAFs exhibited high α-SMA expression and were most prevalent close to tumor foci as opposed to the iCAFs, which were located more distantly from the neoplastic cells within the dense stroma and expressed low α-SMA and high inflammatory mediators and chemokines. Transcription profiling defined a contractile and stroma remodeling phenotype for myCAFs and a secretory phenotype for iCAFs [74]. As a matter of fact, CAFs and activated PSCs are different stromal cell populations in PDAC. Although both cell types share common biomarkers, none of these are specific [74,80]. A clear distinction between CAFs and activated PSCs is still under debate. Several studies over the years revealed a broad functional profile of PSCs by which they contribute to overall tumor progression. However, various attempts to alter or target PSCs and their secretions have largely been unsuccessful [81], underscoring the importance of future investigations into the roles of PSCs in attempts to develop new and effective treatment regimens for PDAC.

**Figure 1:**
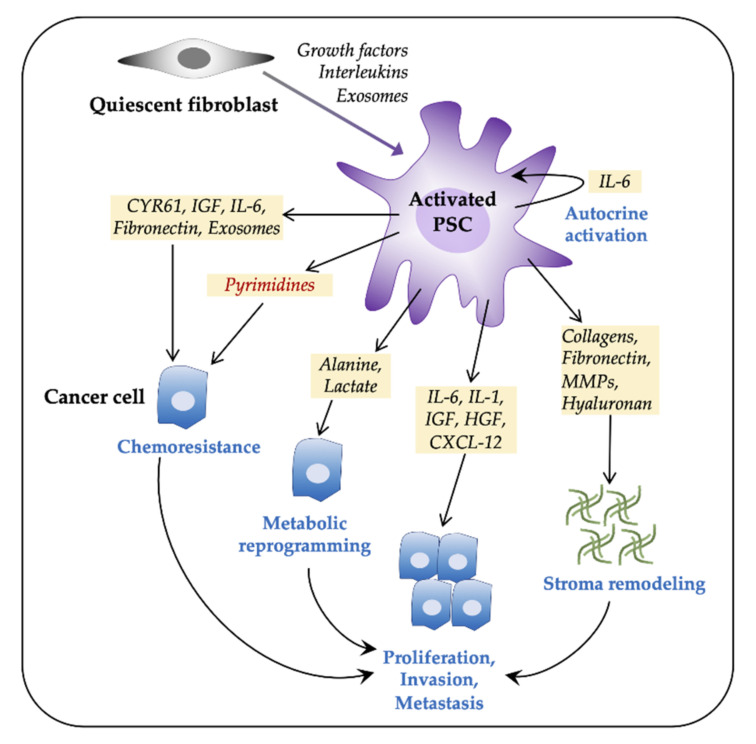
Pancreatic stellate cells (PSCs) facilitate tumor progression in pancreatic ductal adenocarcinoma (PDAC). Pancreatic tumors consist of a large number of activated fibroblasts, which are myofibroblast-like cells and are referred to as PSCs. Fibroblasts are activated by both paracrine and autocrine signals. Activated PSCs promote cancer cell proliferation, invasiveness, metastasis, and chemoresistance via various paracrine, exosome-mediated, or autophagic mechanisms. PSCs contribute to tumor progression by metabolic reprogramming and by extracellular matrix components-mediated stromal remodeling. CXCL12, C-X-C motif chemokine 12; CYR61, cysteine-rich angiogenic inducer 61; HGF, hepatocyte growth factor; IL, interleukin; IGF, insulin-like growth factor; MMPs, matrix metalloproteinases; PDAC, pancreatic ductal adenocarcinoma.

Following the failure of stromal-depletion approaches, the interactions between tumor cells and different stromal components, particularly PSCs, and their multifaceted contributions to PDAC progression have been thoroughly re-assessed [21,66,82]. Among several PSC-induced changes in cancer cells, metabolic reprogramming conducive to tumor growth and reduced chemoresponse are two major changes that are integral to tumor progression (Figure 1). In fact, two fundamental questions have gained particular attention recently, which are (i) how do PSCs help tumor cells to survive and proliferate in a hypoxic, nutrient-poor metabolically challenging environment in PDAC and (ii) to what extent is the poor response to existing therapeutics in PDAC a consequence of metabolic rewiring? In contrast to various studies that have shown that PSCs modulate cancer cell chemosensitivity and also contribute to therapy resistance [23,24,83], it is only recently that PSCs are being explored for their role in metabolic regulation of PDAC, especially as a nutrient supplier [14,22,84].

### 3.1. PSCs Facilitate Tumor Progression

Substantial evidence indicates that stroma forms a supportive niche for pancreatic tumors to grow despite adverse conditions and that PSCs in particular are a significant factor in this process [85,86,87,88]. Tumor cells recruit PSCs via mitogenic and fibrogenic factors, which promote activation, proliferation, migration, and their extracellular matrix (ECM) remodeling capability. For instance, according to Bachem et al. [86], the supernatants from Mia PaCa-2, Panc-1, and SW850 cells stimulate PSCs proliferation and ECM synthesis in a dose-dependent manner. These effects were abrogated by neutralizing antibodies against platelet-derived growth factor (PDGF), fibroblast growth factor 2 [89], and transforming growth factor-β1 (TGF-β1) [90]. Moreover, cancer cells secrete ECM metalloproteinase inducer (EMMPRIN) that promotes the synthesis of matrix metalloproteinase (MMP)-2 by PSCs, which is crucial for degradation of the basement membrane and thereby influence cancer invasion and metastasis [91]. According to Erkan et al., PSCs, once stimulated by cancer cells, remain active via an autocrine periostin loop even under active treatment, sustaining fibrogenic stellate cell activity and tumor progression [92]. Similarly, periostin was expressed strongly in PSCs and in the stroma. Increased expression of periostin was shown to be associated with advanced disease state and reduced survival [93].

Activated PSCs produce high levels of growth factors and inflammatory mediators that maintain their activated phenotype. In particular, inflammation functions as a promoter of tumorigenesis [94] providing tumor cells with survival advantages through the IL-1α signaling cascade, as shown by Tjomsland et al. [95]. PSC-conditioned medium (PSC-CM) affects cancer cell behavior including proliferation, migration, and invasion via a variety of different molecular mechanisms [87,96,97,98,99]. For example, PSCs stimulate invasion and migration of PC cells by production of stromal cell-derived factor-1 (SDF-1) secreted protein acidic and rich in cysteine (SPARC) and matrix metalloproteinases (MMPs) [85,91,100,101,102]. Moreover, upon activation, PSCs acquire a proinflammatory phenotype and secrete proteins in a eukaryotic translation initiation factor eIF4E-dependent manner [103]. According to Lu et al. [104], the PSC-secreted ECM component collagen-I is a major mediator of migration of PC cells via activation of the α2β1 integrin-FAK signaling pathway. Co-culturing of PSCs with cancer cells promotes EMT [105], whereas in vivo co-injection of PSCs with cancer cells in an orthotopic murine model revealed increased primary tumor incidence, tumor size, and distant metastasis [88]. Interestingly, Xu et al. [106] suggest that human PSCs were able to accompany cancer cells to metastatic sites and stimulate angiogenesis. Human PDAC-derived PSCs were also shown to promote cell viability and invasion of Mia PaCa-2 cells in vitro and an increased rate of tumor growth was seen in mice bearing inoculated PSCs and Mia PaCa-2 cells as compared to inoculated Mia PaCa-2 alone [107]. These findings clearly demonstrated reciprocal interactions between PSCs and cancer cells. As such, cancer cells recruit and activate PSCs, which in turn produce a conducive environment to promote local tumor growth and metastatic expansion. However, the precise molecular mechanisms underlying many of these PSC-induced effects on tumor growth and metastasis remain elusive.

### 3.2. PSCs and Gemcitabine Chemoresistance

The dismal clinical outcome of current PDAC chemotherapy is partially linked to chemoresistance that typically exists de novo and/or acquired during exposure of tumors to various chemotherapeutic agents [8]. Despite modest clinical effects, gemcitabine has been the preferred drug for all stages of PDAC since its inception [8,23]. Although the exact mechanisms of gemcitabine chemoresistance are only partly understood [8], biophysical properties of the tumor, in addition to pharmacokinetics and pharmacodynamics of the drug are involved [108,109,110]. The cause of gemcitabine chemoresistance in PDAC is clearly multifactorial, and may be generally classified into four major mechanisms—(i) reduced intracellular transport, (ii) enhanced drug efflux, (iii) dysregulated drug metabolism, and (iv) changes in cellular signaling that negatively affect drug-induced cytotoxicity [23]. In this context, it is noteworthy that despite constituting the major cellular component of PDAC tumors, PSCs were largely ignored in the past for their putative role in the regulation of cancer cell chemosensitivity. It is only recently that PSCs were recognized to modulate tumor cell chemoresponse [23,24,99,111]. PSCs promote chemoresistance to gemcitabine through multiple mechanisms involving the physical barrier mechanisms of the stroma, altered drug bioavailability including a recently suggested drug scavenging effect, and PSC-induced molecular changes in tumor cells [24,83,110,112,113].

#### 3.2.1. Stroma—Biophysical Barrier and Drug Scavenging

With the activation of PSCs, there is a consistent propagation of fibrosis, which is considered a major limiting factor in the delivery of therapeutics to the carcinoma cells. The hypovascular and dense stroma in PDAC is believed to act as a physical barrier to efficient drug delivery [113,114,115,116]. The chemotherapeutic agents are required to cross the blood vessel wall, traverse the extracellular compartment, and enter the cytoplasm of cancer cells to ultimately reach their target sites [117]. The high interstitial fluid pressure (IFP) generally present in PDAC stroma limits the movement of cytotoxic agents from the vasculature to the extracellular compartment [114,118,119]. Rice et al. reported that matrix stiffness in PDAC induced chemoresistance to paclitaxel but not to gemcitabine in vitro, suggesting that environmental rigidity may underlie some aspects of chemoresistance [120]. PDAC stroma is often rich in the ECM component hyaluronan (hyaluronic acid; HA), which is a major contributor to increased IFP thereby limiting drug delivery to cancer cells. Consequently, targeting HA has been considered an attractive target to overcome chemoresistance. Recent studies have shown that enzymatic targeting of HA in PDAC using pegvorhyaluronidase alfa (PEGPH20), depleted stromal HA, reduced IFP, and substantially improved the effects of gemcitabine [114,121].

In recent years, exosomes, extracellular vesicles secreted by CAFs have been implicated in the tumor–stroma crosstalk associated with chemoresistance. Richards et al. found that CAFs exposed to gemcitabine significantly increased exosome release, which subsequently stimulated the expression of the transcription factor Snail, a known chemoresistance-inducing factor, in the recipient tumor cells, causing enhanced proliferation and drug resistance [122]. Furthermore, a study by Fang et al. [123] demonstrated that human CAFs-derived exosomal miRNA, miR-106b, contributes to gemcitabine resistance in PDAC. According to a recent study by Hessmann et al. [112], PSCs entrap active gemcitabine intracellularly, causing drug scavenging and thereby limiting the availability of gemcitabine to tumor cells that may subsequently contribute to the clinical failure of gemcitabine. This finding suggests a novel and alternative mechanism underlying the functional biophysical stroma barrier for drug delivery in PDAC. However, the phenomenon of PSC-induced drug scavenging requires further validations. In contrast to the findings by Hessmann et al. [124], our recent study in human PDAC-derived paired primary co-cultures of PSCs and cancer cells demonstrates that gemcitabine uptake and its intracellular processing in PSCs are significantly lower compared to uptake and processing capacities in primary PC cells and PDAC cell lines.

#### 3.2.2. PSC—Tumor Cell Crosstalk in Induction of Chemoresistance

Multiple lines of evidence implicate interactions between PSCs and cancer cells in the induction of gemcitabine chemoresistance in PDAC [8,23,24,67,88,125,126]. According to Miyamoto et al., PC cells cultured on a fibronectin-coated surface showed a varying degree of increased resistance to gemcitabine, suggesting that ECM proteins are implicated in the induction of chemoresistance [127]. Similarly, our recent study identifies PSC-secreted fibronectin as a chemoresistance-inducing factor in PC cells and that PSCs induce a varying degree of resistance to gemcitabine via activation of MAPK/ERK signaling in seven different human PC cell lines [24]. The study further observed that compared to fibronectin alone, the PSC-CM induced significantly higher chemoresistance, suggesting that other components of the PSC-secretome such as collagens may also contribute to the chemoresistance. Knowledge of the role of different collagens in chemoresistance in PDAC is limited, however, it is known that high gene expression of collagen correlated with drug resistance in for example ovarian and breast cancer cell lines [128,129,130]. Panc-1 cells cultured in 3D collagen have shown increased gemcitabine resistance due to enhanced histone acetylation, possibly affecting gene expression through activation of PI3K/AKT and ERK signaling pathways, leading to increased proliferation despite drug treatment [131,132]. Human PSCs were shown to promote chemoresistance in Mia PaCa-2 cells by downregulation of gemcitabine-induced apoptosis [107]. Similarly, both in vitro and in vivo gene silencing of periostin, a secretory protein exclusively expressed by PSCs, revealed that periostin regulates gemcitabine-induced apoptosis and plays a role in the progression of chemoresistance [133].

Several growth factors and pro-inflammatory components secreted by PSCs have also been shown to induce therapeutic resistance in PDAC. For instance, CAF-secreted insulin-like growth factors (IGF)-1 and -2 promote chemoresistance to gemcitabine in PC cells, whereas pharmacologic inhibition of IGF re-sensitizes PDAC to gemcitabine in vivo [134]. Moreover, Long et al. showed that CAF-derived interleukin (IL)-6 induces STAT3 activation in PC cells, which mediates PDAC chemoresistance [135], whereas Duluc et al. demonstrated a role of the mTOR/4E-BP1 pathway in promoting chemoresistance via autocrine and paracrine IL-6 loop [136]. Neumann et al. demonstrated that direct cell–cell contact and high levels of IL-6 during co-cultures of CAFs and PDAC cell lines correlate with a high degree of chemoresistance [137]. Furthermore, PSCs contribute to PDAC chemoresistance via the release of nitric oxide, IL-1β, and type 1 collagen signaling [92,138,139,140]. According to Wei et al., CAF-secreted SDF-1 (CXCL12) stimulated malignant progression and gemcitabine resistance in PDAC are partially linked to paracrine induction of SATB-1, the special AT-rich sequence-binding protein 1 [141]. Moreover, while PSCs strongly express hepatocyte growth factor (HGF), its receptor c-Met is mainly expressed by cancer cells. Functionally, paracrine HGF from PSCs can activate the c-Met/PI3K/Akt pathway in cancer cells, leading to inhibition of cancer cell apoptosis and induction of chemoresistance to gemcitabine [142]. Co-culture studies of PSCs and PDAC cell lines BxPC-3 and Panc-1 have demonstrated induction of gemcitabine chemoresistance via increased expression of hairy and enhancer of split-1 (HES1), which is also associated with poor prognosis of PDAC patients. The PSC-induced chemoresistance was reversed by HES 1 siRNA and a Notch signaling inhibitor [143].

#### 3.2.3. Intracellular Processing of Gemcitabine in PSCs and Cancer Cells

**Figure 2:**
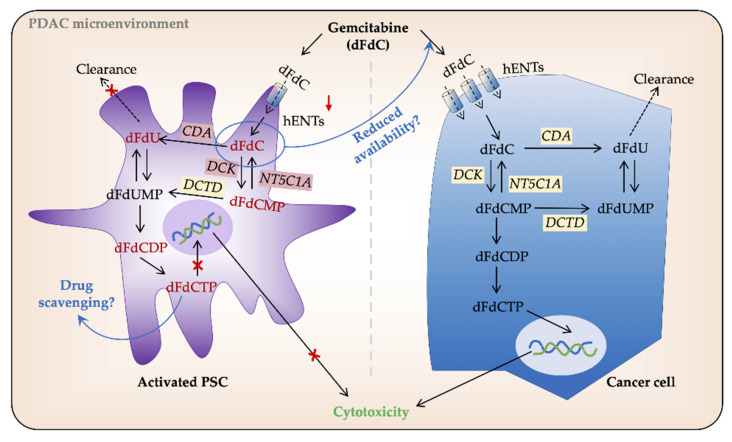
Gemcitabine processing in PSCs and cancer cells. In pancreatic cancer (PC) cells gemcitabine (2′,2′-difluoro-2′-deoxycytidine; dFdC) is transported intracellularly by human equilibrative nucleoside transporters (hENTs). Once inside the cell, its major proportion is inactivated by cytidine deaminase (CDA) and secreted extracellularly. The remaining gemcitabine is activated, mainly by deoxycytidine kinase (DCK), to its subsequent mono-, di-, and tri-phosphate forms dFdCMP, dFdCDP, and dFdCTP, respectively. dFdCTP competes with the substrate for DNA synthesis and induces cell death by apoptosis. Low/no expression of hENTs and other gemcitabine metabolizing enzymes reduce gemcitabine uptake and processing in PSCs. However, a large volume of PDAC tumors is made up of PSCs, and thus, any amount of gemcitabine uptake by PSCs may result in its reduced availability for PC cells. Similarly, intracellular entrapment of dFdCTP by PSCs is speculated to result in drug scavenging. DCTD, deoxycytidylate deaminase; dFdU, 2′,2′-difluoro-2′-deoxyuridine; dFdUMP, 2′,2′-difluoro-2´-deoxyuridine monophosphate; NT5C1A, 5′-nucleotidase cytosolic 1A; PSC, pancreatic stellate cell.

Gemcitabine (dFdC) is a deoxynucleoside analog that requires transmembrane transport and intracellular activation by phosphorylation to generate its active form dFdCTP that ultimately inhibits DNA synthesis and induces apoptosis [8]. The pharmacokinetics of gemcitabine in tumor cells is regulated by human equilibrative nucleoside transporters (hENTs)-mediated uptake, activation by deoxycytidine kinase (DCK), and inactivation by cytidine deaminase (CDA), deoxycytidylate deaminase (DCTD), and cytosolic 5′-nucleotidases (NT5C1A) (Figure 2). Altered expression of these enzymes may lead to reduced gemcitabine-induced cytotoxicity, and thereby chemoresistance [8]. The nucleoside transporters hENT1 and hCNT3 are crucial mediators of gemcitabine uptake in PC cells, and their downregulation causes chemoresistance [8]. According to Hesler et al. [113], PSCs are a source of cysteine-rich angiogenic inducer 61 (CYR61), which promotes gemcitabine resistance in PC cells by downregulating nucleoside transporters hENT1 and hCNT3, subsequently reducing drug uptake. Moreover, PSC-secretomes affect cancer cell chemosensitivity. For example, the conditioned medium from PSCs and other fibroblasts protects PC cells from gemcitabine-induced cytotoxicity because PSCs secrete deoxycytidine, which competes with gemcitabine processing by DCK in PC cells, thereby promoting chemoresistance [83].

Gemcitabine inactivating enzyme NT5C1A negatively regulates gemcitabine activity. Interestingly, murine PSCs displayed high intracellular gemcitabine levels both in vitro and in vivo, which was supported by lower levels of NT5C1A [112]. Similarly, the same group recently showed that NT5C1A is overexpressed in both murine and human PDAC and that in mice, NT5C1A mediates gemcitabine resistance by decreasing the amounts of intracellular dFdCTP [144]. In addition, a co-culture experiment with conditioned media from NT5C1A expressing PSCs improved gemcitabine efficacy in tumor cells [144]. Our recent study demonstrated high and variable NT5C1A expression in PDAC cell lines including BxPC-3, Mia PaCa-2, and Panc-1, and in human PDAC-derived primary PC cells, whereas NT5C1A expression was undetectable in PSCs derived from the same tumors. Of note, NT5C1A expression did not correlate with gemcitabine chemosensitivity in this model system as the PC cells were far more chemosensitive than PSCs despite high NT5C1A expression [124]. NT5C1A could thus offer a potential therapeutic target for PDAC treatment, however, further investigations to this end are clearly warranted. Moreover, PSCs are intrinsically resistant to the cytotoxic actions of gemcitabine. This finding could at least partially be explained by the very low or non-existent protein expression of hENT1 and DCK in human PDAC-derived primary PSCs as compared with PC cells [124]. Lack of hENT1 and DCK causes failure of transport and intracellular activation of gemcitabine, thus causing the observed chemoresistance in PSCs. Our study further revealed a negative correlation between gemcitabine sensitivity in terms of IC_50_ values and the intracellular levels of active metabolites of gemcitabine, dFdCDP, and dFdCTP, both individually and in combination [124]. Moreover, our data demonstrate that in the cancer cells there is a strong correlation between gemcitabine sensitivity and the protein expression ratio hENT1 × DCK/CDA × DCTD [124]. This ratio could thus serve as a novel, informative and predictive marker for gemcitabine sensitivity in PDAC. Altogether these findings suggest that PSCs affect PDAC chemoresistance in multiple and complex ways that are yet to be fully explained.

### 3.3. PSCs Facilitate Metabolic Rewiring in PDAC

**Figure 3:**
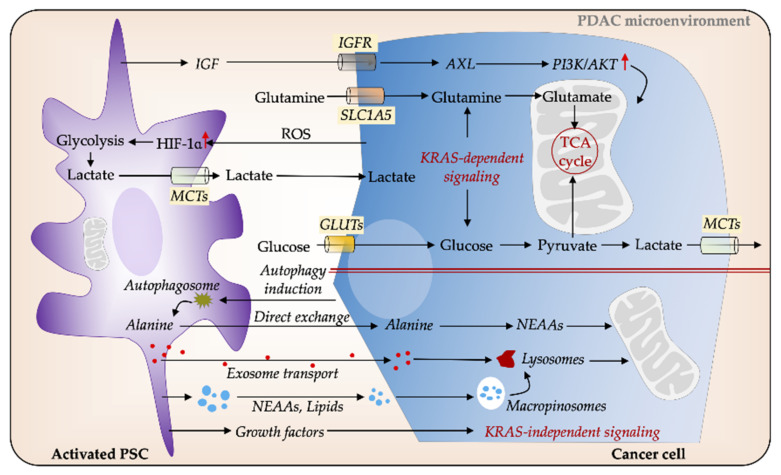
PSCs facilitate metabolic reprogramming in PDAC. Cancer cells secrete reactive oxygen species (ROS), which induces glycolysis in PSCs, thereby increasing the release of nutrients such as lactate, which in turn feed the energy demands of cancer cells. Several cytokines and signaling pathways mediate metabolic interactions between PSCs and PC cells via KRAS-dependent pathways. Insulin-like growth factor (IGF) increases mitochondrial respiration in cancer cells via IGFR/AXL axis. Glucose and glutamine are major sources of carbon for cancer cells. PSCs-secreted non-essential amino acids (NEAAs), such as autophagy-induced alanine, can serve as an alternative energy source to fuel cancer cells. Cancer cells can obtain nutrients from extracellular proteins for supporting their growth through upregulated macropinocytosis. In addition, PSCs-derived growth factors (GFs) and exosomes play a pivotal role in cancer cell metabolic balance. GLUT, glucose transporter; MCT; monocarboxylate transporter; PDAC, pancreatic ductal adenocarcinoma; PSC, pancreatic stellate cell.

The environmental stress within PDAC imposes nutrient shortage in cancer cells. Despite diverse mechanisms promoting extracellular glucose acquisition in cancer cells via the Warburg or the reverse Warburg effect, enhanced glucose metabolism alone cannot completely compensate for the increased energetic and biosynthetic demands of tumor cells [15,30,49,145,146]. Metabolic rewiring in cancer cells and stromal components of PDAC enables access to the recycling of nutritional substrates and alternate fuel sources, in order to sustain tumor growth and survival [18,84]. A detailed understanding of the PSC-tumor interplay may therefore offer new targets for future treatment strategies (Figure 3).

PSCs are postulated to be the alternate energy supplier for tumor cells in PDAC. PSCs are to a significant degree able to reprogram the metabolic machinery of PDAC, in particular, the metabolic crosstalk between PSCs and tumor cells that facilitates tumor progression and invasiveness [14,22,147]. Conversely, PC cells could force PSCs to provide the cancer cells with energy and nutrients. PSCs surrounding tumor cells also undergo a metabolic transition into a phenotype that displays characteristics corresponding to the Warburg effect. Compared to quiescent fibroblasts, activated PSCs take up more glucose and produce more lactate, which subsequently is actively taken up by tumor cells to support their growth [148]. PSC-derived non-essential amino acids (NEAAs) also provide nutrients to feed PC cells.

Recently, PSCs were shown to regulate tumor cell metabolic activities through autophagic alanine secretion [22]. Autophagy is also required for the activation of PSCs, in addition to being associated with the growth and progression of pancreatic tumors in mice [149]. Tumor cells increase autophagy in PSCs via unknown mechanisms and stimulate alanine secretion, which serves as an alternative carbon source of pancreatic tumors. The PSC-secreted alanine is taken up by tumor cells, thus outcompeting glucose and glutamine to support the tricarboxylic acid (TCA) cycle in order to produce NEAAs and lipids [14,22]. Furthermore, PSC-derived exosomes provide another source of alanine to fuel cancer cells and the resulting metabolic remodeling was described to be independent of KRAS mutation [150]. Collectively, the tumor–stromal metabolic crosstalk directs PSCs to supply energy and assist tumor cells to thrive in the hostile, hypoxic, and nutrient-poor PDAC environment.

Hypoxia-inducible factor (HIF)-1α, a protein expressed under hypoxic conditions, is overexpressed in PDAC and associated with poor prognosis [151,152,153,154]. Hypoxia increases survival, proliferation, EMT, and invasiveness of PC cells and promotes resistance to therapy through HIF-1α -dependent and -independent mechanisms [155,156,157,158,159]. Moreover, tumor cells stabilize HIF-1α in PSCs by increasing ROS production to increase glycolysis, leading to the formation of a “pseudo-hypoxic” environment for PSCs [160]. The metabolic interplay between PSCs and cancer cells is considered a consequence of genetic mutations combined with a comprehensive paracrine signaling network [14,15,161]. Oncogenic KRAS signaling has been reported to play a predominant role in multiple aspects of PDAC metabolism, including adaptive metabolic responses and PSC-tumor cell interplay [14,162,163]. It is becoming increasingly apparent that KRAS mutations manipulate signaling in both tumor cells and neighboring PSCs and influence the metabolic interactions between the two cell types [164]. KRAS promotes sonic hedgehog secretion from PC cells, which causes activation of PSCs to induce secretion of various cytokines such as IGF1, GAS6, and GM-CSF. Subsequently, PSCs send reciprocal feedback signals to PC cells via the IGF1R/AXL axis, which activates downstream PI3K-AKT phosphorylation. This leads to increased mitochondrial respiratory capacity in PC cells and subsequently elevated oxygen availability under hypoxia [164].

## 4. Metabolic Reprogramming and Gemcitabine Chemoresistance in PDAC: An Evolving Concept

The putative molecular relations between metabolic reprogramming and gemcitabine chemoresistance in PDAC is a new field of investigation and still only scantily understood. Increasing evidence indicates that gemcitabine resistance is related to the metabolism of glucose, amino acids, and lipids [165]. In addition, metabolic profiling revealed an obvious difference in the metabolome between gemcitabine-sensitive and gemcitabine-resistant PC lines. In particular, gemcitabine-resistant PC cells displayed reduced glutamine and proline levels, in addition to elevated aspartate, hydroxyproline, creatine, and creatinine levels in gemcitabine-resistant cells compared to gemcitabine-sensitive cells [166].

PDAC is known for enhanced HIF-1α levels, which maintain a functional relationship between tumor cells and stromal fibroblasts by upregulating the expression and secretion of Sonic hedgehog [167]. Interestingly, HIF-1α is recently being pursued as a potential target of PDAC treatment as chemoresistant pancreatic cancer cells were shown to exhibit increased glycolysis [168], which is partly regulated by HIF-1α [169]. MUC1 mucin is reported to facilitate growth permissive metabolic alterations in the hypoxic PDAC environment via stabilization and activation of HIF-1α [170]. In addition, MUC1 and HIF-1α signaling crosstalk induces anabolic glucose metabolism and impart gemcitabine resistance in PC [171]. Mechanistically, increased glycolytic flux leads to glucose addiction in cancer cells and a corresponding increase in pyrimidine biosynthesis to enhance the intrinsic levels of deoxycytidine triphosphate (dCTP), which diminish the effective levels of gemcitabine through molecular competition. Moreover, targeting HIF-1α or de novo pyrimidine biosynthesis increases the efficacy of gemcitabine [171,172].

According to He et al. [173], hypoxia-induced ERK1/2 phosphorylation causes HIF-1α activation, which contributes to ABCG2-mediated gemcitabine chemoresistance in PC cells. Inhibition of ERK1/2 and HIF-1α was shown to increase the gemcitabine sensitization. Similarly, our recent findings demonstrated that PSC-secreted fibronectin mediates gemcitabine sensitivity in PC cells via ERK1/2 signaling [24]. Furthermore, fructose-1,6-bisphosphatase inhibits the activation of ERK and bypasses gemcitabine resistance in PDAC by blocking the IQGAP1/ERK1/2 signaling pathway independent of its enzymatic activity [174]. Interestingly, gemcitabine-resistant PC cells exhibit increased glucose uptake and glycolysis, and downregulated hENT1, whereas hENT1 overexpression experiments revealed that hENT1 negatively regulates glycolysis through HIF-1α and c-Myc and their target genes and reverses gemcitabine chemoresistance in PDAC [175].

The role of the amino acid and lipid metabolisms in the regulation of PDAC chemoresistance is far less known compared to glucose metabolism. Glutamine, a key regulator of KRAS-mediated metabolic rewiring in PDAC, has gathered attention in recent years. Glutamine-dependent mTOR activation contributes to increased glycolysis and gemcitabine resistance [176]. Oncogenic KRAS-induced NRF2 upregulates glutaminolysis and promotes gemcitabine resistance, whereas inhibition of the glutamine metabolic pathway sensitizes chemoresistant pancreatic cells to gemcitabine [177,178]. In PDAC lipid metabolism, fatty acid synthase (FASN) plays a crucial role as overexpressed FASN in PC cells upregulates pyruvate kinase M2, which promotes glycolysis and gemcitabine resistance [179]. In addition, Orlistat, a FASN inhibitor was shown to increase gemcitabine sensitivity in mouse models with orthotopic PC implants [180]. Moreover, in addition to glucose, amino acids, and lipids metabolism, autophagy, which is required for glutamine metabolism in PDAC is considered to play a role in chemoresistance. Autophagy is induced by both nutrient limitation and gemcitabine. Glutamine deprivation in PDAC activates autophagy, which inhibits apoptosis and contributes to gemcitabine resistance [181].

These findings indicate that to overcome gemcitabine resistance in PDAC and effectively eliminate drug resistance, combined strategies involving modulation of both glycolytic and mitochondrial pathways might prove beneficial. A recent study by Masoud et al. [182] revealed that targeting mitochondrial respiratory complex 1 using its inhibitor, phenformin, overcomes gemcitabine chemoresistance in high OXPHOS PDAC tumors. Different metabolic pathways are significantly altered in drug-resistant tumor cells and if the metabolic profile is well established, targeting these pathways could significantly improve therapeutic outcome. In general, gemcitabine chemoresistance has complex relations with PDAC metabolism, and insight into these mechanisms may pave the way towards more efficient chemotherapy.

## 5. Conclusions

Unlike many other solid tumors, pancreatic tumors are extremely chemoresistant and highly metabolically active. Tumor progression in PDAC is supported by stroma-induced metabolic reprogramming, which creates a growth-permissive environment and affects the biological behavior of PC cells. PSCs are the principal stroma-producing cells and increasing evidence suggests that multi-faceted interactions between PC cells and PSCs are pivotal to PDAC progression. Of the many effects of PSCs on PC cells, mechanisms by which PSCs provide nutrients to tumor cells in the hostile conditions of the TME, and their contribution towards the chemoresistance phenotype of PDAC, offer potential avenues for developing new therapeutics.

## Abbreviations

ABCG2	ATP-binding cassette super-family G member 2
ATP	Adenosine triphosphate
CAF	Cancer-associated fibroblast
CDA	Cytidine deaminase
CYR61	Cysteine-rich angiogenic inducer 61
DCK	Deoxycytidine kinase
DCTD	Deoxycytidylate deaminase
ECM	Extracellular matrix
EMT	Epithelial-mesenchymal transition
EMMPRIN	Extracellular matrix metalloproteinase inducer
FAK	Focal adhesion kinase
FASN	Fatty acid synthase
GAS6	Growth arrest—specific 6
GISS	Growth-induced solid stress
GM-CSF	Granulocyte-macrophage colony-stimulating factor
HA	Hyaluronic acid
HIF-1α	Hypoxia-inducible factor (HIF)-1α
hENT-1	Human nucleoside transporter 1
iCAF	Inflammatory cancer-associated fibroblast
IFP	Interstitial fluid pressure
IGFR/AXL	Insulin-like growth factor/AXL receptor tyrosine kinase
IQGAP1	Ras GTPase-activating-like protein
myCAF	Myofibroblastic cancer-associated fibroblast
MMP	Matrix metalloproteinases
mTOR	Mechanistic target of rapamycin
NEAA	Non-essential amino acids
NT5C1A	Cytosolic 5´-nucleotidases 1A
OXPHOS	Oxidative phosphorylation
PC	Pancreatic cancer
PDAC	Pancreatic ductal adenocarcinoma
PEGPH20	Pegvorhyaluronidase alfa
PSC	Pancreatic stellate cell
PSC-CM	PSC-conditioned medium
ROS	Reactive oxygen species
SATB-1	Special AT-rich sequence-binding protein-1
TCA	Tricarboxylic acid
TME	Tumor microenvironment
4E-BP1	Eukaryotic translation initiation factor 4E binding protein 1
